# Screening for retinopathy of prematurity in North China

**DOI:** 10.1186/s12886-022-02470-3

**Published:** 2022-06-06

**Authors:** Li Li, Yanlin Gao, Wei Chen, Mei Han

**Affiliations:** Tianjin Eye Hospital, Tianjin Key Laboratory of Ophthalmology and Vision Science, Nankai University Eye Hospital, Clinical College of Ophthalmology, Tianjin Medical University, 4, Gansu Road, Heping District, Tianjin, 300020 People’s Republic of China

**Keywords:** Retinopathy of prematurity, Gestational age, Birth weight, Screening criteria

## Abstract

**Purpose:**

To analyze the incidence and severity of retinopathy of prematurity (ROP) in north China, and to evaluate the effectiveness of different ROP screening criteria.

**Patients and methods:**

The screening data of premature infants were collected from 2016 to 2021. The severity of ROP was graded according to the International Classification of Retinopathy of Prematurity (2005). And the treatment for ROP followed the Early Treatment for Retinopathy of Prematurity Cooperative Group. The effects of gestational age (GA) and birth weight (BW) on the incidence and severity of ROP were evaluated. The screening data were also analyzed using different ROP screening guidelines.

**Results:**

A total of 4069 infants underwent ROP screening, and 728 infants (17.9%) were diagnosed with ROP. Of those, 78 infants (1.9%) received treatments. Gestational age and BW showed significant differences between infants with and without ROP (29.1 ± 2.1w vs. 32.9 ± 2.6w, *p* < 0.001; 1362.7 ± 427.3 g vs. 1751.9 ± 509.4 g, *p* < 0.001; respectively). Fifty-six infants (7.69%), 188 infants (25.82%), and 104 infants (14.29%) in all infants with ROP would have been missed according to the China, USA, and UK screening guidelines respectively. If GA ≤ 33 weeks and/or BW ≤ 2100 g were considered as screening criteria, only one infant (0.14%) with critical systemic illness was missed diagnosed with severe ROP.

**Conclusion:**

Gestational age and BW are major risk factors for the incidence and severity of ROP. And the incidence and treatment rate of ROP in Tianjin is similar to that reported in the other regions of China. Modified ROP screening criteria were considered to be more effective in Tianjin.

## Introduction

Retinopathy of prematurity (ROP) is characterized by abnormal retinal neovascularization and can cause severe visual disability in childhood. The survival rate of premature infants with a lower birth weight (BW) has been on the rise because of the benefits of medical progress. The incidence of ROP had increased and it becomes the leading cause of childhood blindness in developing countries, including China [1, 2].

Gestational age (GA) and BW are identified as major risk factors for the development of ROP. Different ROP screening criteria were carried out in different countries mainly based on BW and gestational age (GA). In 2014, the Chinese Ophthalmological Society has published the ROP screening guideline as followed: infants with a GA < 32 weeks and/or BW < 2000 g, or infants who were suspected to be a risk of ROP (such as infants who received long-term oxygen supplementation or infants with serious systemic diseases) [3]. According to the USA screening guidelines, preterm infants with BW < 1500 g or GA < 30 weeks as well as other serious clinical courses should be screened [4]. According to the UK screening guidelines, infants with BW ≤ 1500 g or GA < 32 weeks as well as other unstable illnesses require fundus examination [5].

In this study, we collected the data of premature infants enrolled in the ROP screening from 2016 to 2021 in Tianjin, China. We analyzed the demographic characteristics, incidence, potential risk factors, and proportions of infants developing different stages of ROP. Based on the BW and GA, we evaluated the effectiveness of different ROP screening criteria and wanted to explore the most appropriate screening standard in Tianjin.

## Patients and methods

### Patients and data collection

This was a retrospective study of infants who received ROP screening in Tianjin Eye Hospital from January 2016 to December 2021. All subjects were diagnosed in the Tianjin Eye Hospital, Tianjin, China. This study adhered to the tenets of the Declaration of Helsinki and was approved by the Ethics Committee of Tianjin Eye Hospital. Written informed consent was obtained from all infants’ parents or guardians. All methods were carried out under relevant guidelines and regulations. BWs and GAs of infants were recorded at each examination.

### Screening criteria

(1) the examinations were carried out according to the ROP guidelines recommended by the Chinese Ophthalmological Society in 2014: [3] infants with a GA < 32 weeks and/or BW < 2000 g, or infants who were suspected to be a risk of ROP (such as infants who received long-term oxygen supplementation or infants with serious systemic diseases). (2) The timing of first examinations is postnatal age of 4–6 weeks or postmenstrual age of 31–32 weeks. (3) the infants with no ROP sign at the first examination were evaluated every 2–3 weeks until retinal vascularization was completed or postmenstrual age of 45 weeks. If ROP was detected, the examinations were performed weekly.

### Fundus examinations and diagnosis

Pupils were dilated with a combination of 0.5% tropicamide and 0.5% phenylephrine eye drops (Mydrin, Santen Pharmaceutical, Osaka, Japan). After topical anesthesia (proparacaine; Alcon Laboratories, Inc., Fort Worth, TX) instillation, fundus examinations were performed by an experienced Ophthalmologist using the RetCam III digital camera (Clarity Medical Systems, USA). The stages of ROP were determined based on the International Classification of Retinopathy of Prematurity (2005) [6]. If different stages appeared in the same eye, the most serious stage of ROP was recorded. The indication of treatment, including type 1 ROP and aggressive posterior ROP (AP-ROP), followed the Early Treatment for Retinopathy of Prematurity (ETROP) Study [7]. Treatment was carried out in 72 h when type 1 ROP and AP-ROP were detected.

### Statistics

The GA and BW of infants were analyzed using the Mann–Whitney U test or Kruskal–Wallis H test according to the different grouping methods. The gender and multiple births were analyzed using Fisher exact test. The statistical analyses were performed with SPSS (version 19.0; SPSS Inc., Chicago, IL). *P* < 0.05 was considered statistically significant.

## Results

### Subject characteristics

A total of 4278 premature infants meeting the screening criteria were admitted from January 2016 to December 2020. However, 209 infants (4.9%) were excluded due to loss to follow-up. Thus, 4069 infants (95.1%) were enrolled in this study. Of these, 393 infants (9.7%) were believed by their pediatricians to be a risk of ROP (such as serious systemic diseases and long-term oxygen supplementation). The mean GA was 32.3 ± 2.6 weeks, and the mean BW was 1751.9 ± 509.4 g. Two thousand one hundred and sixty-three (53.2%) infants were males, 3102 (76.2%) infants were singletons, and 967 (22.1%) of multiple deliveries.

Retinopathy of prematurity screening data was shown in Table 1. A total of 728 infants (17.9%) were diagnosed with ROP. Gestational age and BW showed significantly different between infants with and without ROP (29.1 ± 2.1 w vs. 32.9 ± 2.6 w, *p* < 0.001; 1362.7 ± 427.3 g vs. 1751.9 ± 509.4 g, *p* < 0.001; respectively). Gender and multiple births showed no differences between infants with and without ROP. Furthermore, logistic regression showed that lower GA and lower BW were independent risk factors for ROP (*p* < 0.001; *p* < 0.001; respectively) (Table 2).

**Table 1:** Characteristics of infants

	No ROP	ROP	*P* value
Number (%)	3341 (82.1)	728 (17.9)	
GA, mean ± SD, weeks	32.9 ± 2.6	29.1 ± 2.1	< 0.001
BW, mean ± SD, g	1751.9 ± 509.4	1362.7 ± 427.3	< 0.001
Gender (male: female)	1772: 1569	387: 341	0.904
Multiple birth	789	158	0.387

**Table 2:** Logistic regression analysis of Gestational age, birth weight, gender and multiple birth as a risk for ROP

Factors		Odds Ratio (95% CI)	*P* value
Gestational age		0.817 (0.775–0.862)	< 0.001
Birth weight		0.999 (0.999–1.000)	< 0.001
Gender (vs. male)	female	0.979 (0.903–1.061)	0.606
Multiple birth (vs. singleton)	multiple deliveries	1.086 (0.895–1.318)	0.405

### Numbers and proportions of infants developing different stages of ROP

**Fig. 1:**
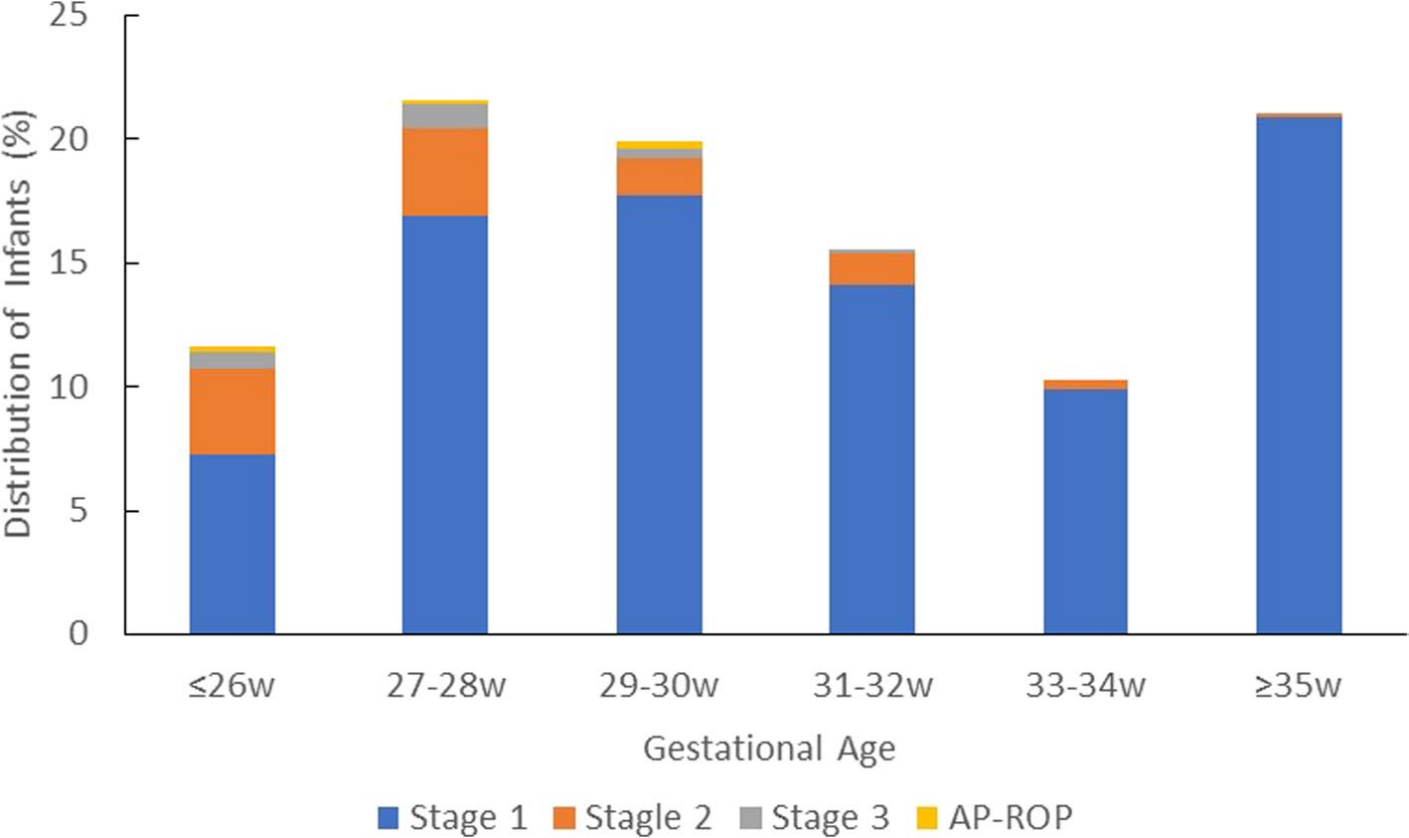
Distribution of infants with retinopathy of prematurity according to gestational age

**Fig. 2:**
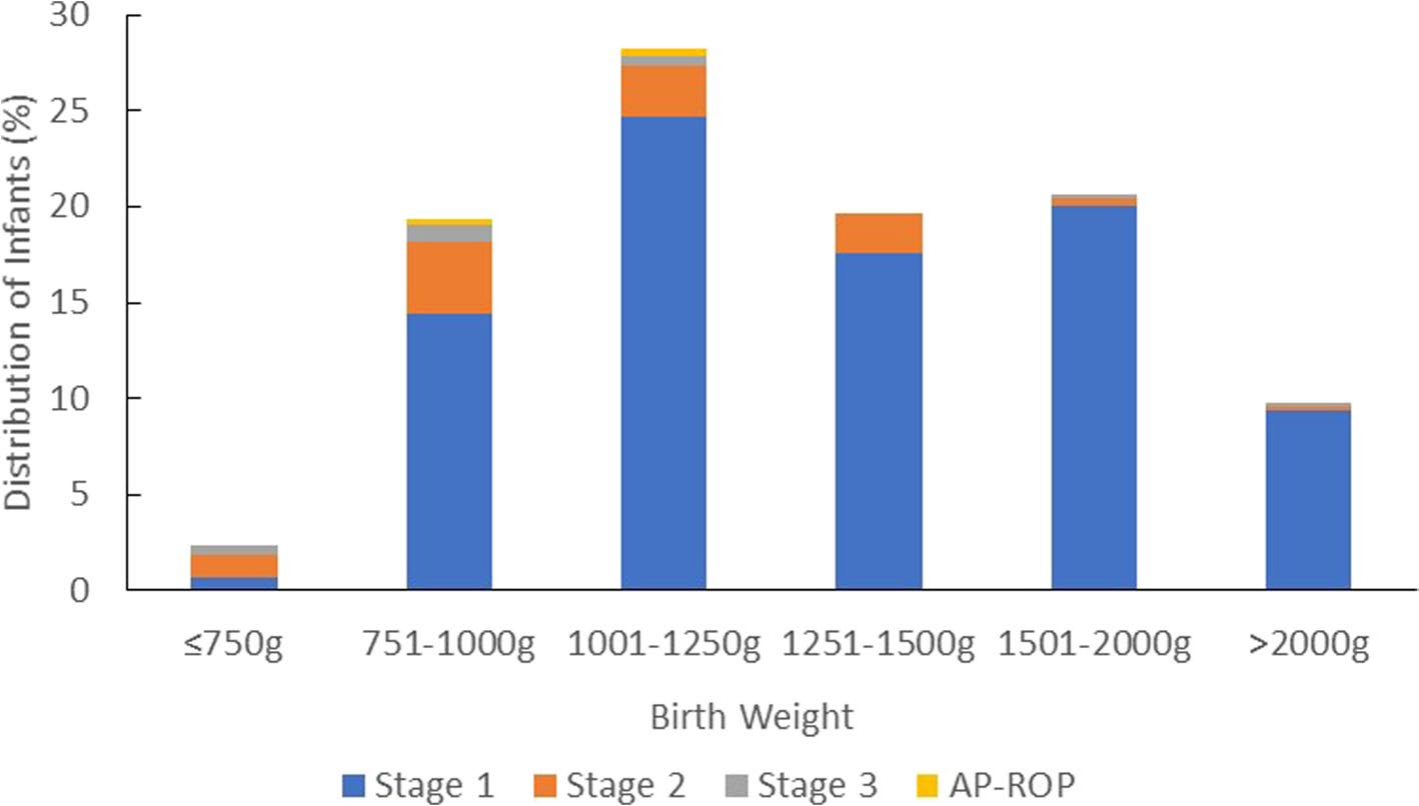
Distribution of infants with retinopathy of prematurity according to birth weight

In general, the proportion of infants developing ROP increased with lower BW and GA (Table 3, Fig. 1, and Fig. 2). Lower GA was a risk factor for ROP, as 58.0% (242/417) of infants with GA ≤ 28 weeks and 22.8% (500/2189) with GA ≤ 32 weeks developed ROP. Lower BW was also a risk factor for ROP, as 66.7% (158/237) of infants with BW ≤ 1000 g and 35.7% (507/1421) of infants with BW ≤ 1500 g developed ROP. Stage 1 of ROP was mostly detected in infants with BW between 1001 and 1250 g or GA ≥ 35 weeks. Stage 2 and Stage 3 of ROP were detected in the infant with BW between 751 and 1000 g or GA between 27 and 28 weeks. AP-ROP was only detected in infants with BW ≤ 1250 g or GA ≤ 30 weeks.

**Table 3:** Numbers and proportions of infants developing different stages of ROP

	No ROP, N (%)	Total ROP, N (%)	ROP Stage 1, N (%)	ROP Stage 2, N (%)	ROP Stage 3, N (%)	AP-ROP, N (%)	Total, N
BW, g							
≤ 750	2 (10.5)	17 (89.5)	5 (26.3)	9 (47.4)	3 (15.8)	0 (0)	19
751–1000	77 (35.7)	141 (64.7)	105 (48.2)	27 (0.9)	7 (3.2)	2 (9.2)	218
1001–1250	279 (57.5)	206 (42.5)	180 (37.1)	19 (3.9)	4 (0.8)	3 (0.6)	485
1251–1500	556 (79.5)	143 (20.5)	128 (18.3)	15 (2.1)	0 (0)	0 (0)	699
1501–2000	1428 (90.5)	150 (9.5)	146 (9.3)	3 (0.2)	1 (0.1)	0 (0)	1578
> 2000	999 (93.4)	71 (6.6)	68 (6.4)	2 (0.2)	1 (0.1)	0 (0)	1070
Total	3341 (82.1)	728 (17.9)	632 (15.5)	75 (1.8)	16 (0.4)	5 (0.1)	4069
GA, weeks							
GA ≤ 26w	13 (13.3)	85 (86.7)	53 (54.1)	25 (25.5)	5 (5.1)	2 (2)	98
27-28w	162 (50.8)	157 (49.2)	123 (38.6)	26 (8.2)	7 (2.2)	1 (0.3)	319
29-30w	509 (77.8)	145 (22.2)	129 (19.7)	11 (1.7)	3 (0.5)	2 (0.3)	654
31-32w	1005 (89.9)	113 (10.1)	103 (9.2)	9 (0.8)	1 (0.1)	0 (0)	1118
33-34w	1037 (93.3)	75 (6.7)	72 (6.5)	3 (0.3)	0 (0)	0 (0)	1112
≥ 35w	615 (80.1)	153 (19.9)	152 (19.8)	1 (0.1)	0 (0)	0 (0)	768
Total	3341 (82.1)	728 (17.9)	632 (15.5)	75 (1.8)	16 (0.4)	5 (0.1)	4069

### Incidence and characteristics of No ROP, Mild ROP, and severe ROP

Incidence of no ROP, mild ROP, and severe ROP was shown in Table 4. The proportion of infants with BW ≤ 1000 g and BW ≤ 1500 g who developed severe ROP was 15.6% (37/237) and 5.2% (74/1412), respectively. The proportion of infants with GA ≤ 28 weeks and GA ≤ 32 weeks who developed severe ROP was 11.8% (49/417) and 3.3% (73/2189), respectively. The incidence of severe ROP was 47.4% in infants with BW ≤ 750 g and 23.5% in infants with GA ≤ 26 weeks. Incidence of severe ROP was 0.1% in infants with BW ≥ 2000 g and GA ≥ 35 weeks.

**Table 4:** Incidence of No ROP, mild ROP, and severe ROP

	No ROP, N (%)	Mild ROP, N (%)	Severe ROP, N (%)	Total ROP, N (%)	Total, N
BW, g					
≤ 750	2 (10.5)	8 (42.1)	9 (47.4)	17 (89.5)	19
751–1000	77 (35.3)	113 (51.8)	28 (12.8)	141 (64.7)	218
1001–1250	279 (57.5)	180 (37.1)	26 (5.4)	206 (42.5)	485
1251–1500	556 (79.5)	132 (18.9)	11 (1.6)	143 (20.5)	699
1501–2000	1428 (90.5)	147 (9.3)	3 (0.2)	150 (9.5)	1578
> 2000	999 (93.4)	70 (6.5)	1 (0.1)	71 (6.6)	1070
Total	3341 (82.1)	650 (16.0)	78 (1.9)	728 (17.9)	4069
GA, weeks					
GA ≤ 26w	14 (14.3)	61 (62.2)	23 (23.5)	84 (85.7)	98
27-28w	163 (51.1)	130 (40.8)	26 (8.2)	156 (48.9)	319
29-30w	507 (77.5)	131 (20.0)	16 (2.4)	147 (22.5)	654
31-32w	1007 (90.1)	103 (9.2)	8 (0.7)	111 (9.9)	1118
33-34w	1036 (93.2)	73 (6.6)	3 (0.3)	76 (6.8)	1112
≥ 35w	614 (79.9)	153 (19.9)	1 (0.1)	154 (20.1)	768
Total	3341 (82.1)	650 (16.0)	78 (1.9)	728 (17.9)	4069

Gestational age showed a significant difference between no ROP, mild ROP, and severe ROP (*P* < 0.001). Birth weight showed a significant difference between no ROP, mild ROP, and severe ROP (*P* < 0.001). Lower GA and BW significantly correlated with the severity of ROP (Table 5).

**Table 5:** Characteristics of No ROP, mild ROP, and severe ROP

	No ROP	Mild ROP	Severe ROP	*P* value
Number	3341	650	78	
GA, mean ± SD, weeks	32.9 ± 2.6	30.0 ± 2.7	27.8 ± 3.0	< 0.001
BW, mean ± SD, g	1751.9 ± 509.4	1421.1 ± 557.7	1158.4 ± 441.6	< 0.001

### Infants needing treatment

Seventy-eight infants (1.9%) with severe ROP underwent treatments. The mean GA was 27.8 ± 3.0 weeks, and the mean BW was 1158.4 ± 435.6 g. Stage 4 and stage 5 of ROP were not detected in the screening infants. Five infants were diagnosed with AP-ROP. Of those, 4 infants underwent intravitreal injection of anti-vascular endothelial growth factor (VEGF) inhibitors and laser photocoagulation therapy. One infant was diagnosed with viral encephalitis by Neurologists and was advised to examine the fundus. Finally, the parents abandoned the ROP treatment because of severe encephalitis. Excluding the infants with AP-ROP, 73 infants with severe ROP underwent intravitreal injection of anti-VEGF inhibitors. Of those, 53 infants underwent intravitreal injection only once, 12 infants underwent intravitreal injection twice, and 8 infants underwent intravitreal injections of both eyes in the same surgery.

### The effectiveness of the different ROP screening criteria

According to the different ROP screening criteria, the numbers of infants missed diagnosed with ROP were shown in Table 6. Using the current China ROP screening guideline, 56 infants (7.69%) of 728 infants with ROP would have been missed from the ROP screening. Of those, 54 infants (7.42%) were diagnosed with mild ROP and 2 infants (0.27%) were diagnosed with severe treatment-requiring ROP. If the USA screening guideline had been chosen, 188 infants (25.82%) in all infants with ROP would have been missed. Of those, 183 infants (25.14%) were diagnosed with mild ROP and 5 infants (0.69%) were diagnosed with severe ROP. According to the UK screening guideline, 104 infants (14.29%) in all infants with ROP would have been missed. Of those, 101 infants (13.87%) were diagnosed with mild ROP and 3 infants (0.41%) were diagnosed with severe ROP. If BW ≤ 2100 g or GA ≤ 33 weeks were chosen as screening criteria, 35 infants (4.81%) in all infants with ROP would have been missed. Of those, 34 infants (4.67%) were diagnosed with mild ROP and 1 infant (0.14%) was diagnosed with severe ROP. This missed infant was born with BW equal to 2720 g and at GA older than 36 weeks. The infants were diagnosed with pneumonia and were given supplemental oxygen treatment. On the other side, additional 302 infants needed to be examined according to the screening criteria of BW ≤ 2100 g or GA ≤ 33 weeks.

**Table 6:** The rate of missed diagnosis of ROP, sensitivity and specificity according to the different screening criteria

Screening Criteria	Infants Missed With Mild ROP, N (%)	Infants Missed With Severe ROP, N (%)	Total Infants Missed With ROP, N (%)	Sensitivity (%)	Specificity (%)	Positive predictive value (%)	Negative predictive value (%)
China Screening Guideline (BW < 2000 g or GA < 32w)	54 (7.42)	2 (0.27)	56 (7.69)	92.3	98.3	92.30	98.32
USA Screening Guideline (BW < 1500 g or GA < 30w)	183 (25.14)	5 (0.69)	188 (25.82)	74.2	93.9	72.58	94.35
UK Screening Guideline (BW ≤ 1500 g or GA < 32w)	101 (13.87)	3 (0.41)	104 (14.29)	85.2	96.9	85.64	96.77
BW ≤ 2000 g or GA ≤ 30w	65 (8.93)	3 (0.41)	68 (9.34)	90.7	98.0	90.66	97.96
BW ≤ 1750 g or GA ≤ 32w	86 (11.81)	2 (0.27)	88 (12.09)	87.9	97.4	87.91	97.37
BW ≤ 1750 g or GA ≤ 30w	134 (18.41)	4 (0.55)	138 (18.96)	81.0	99.6	98.01	96.02
BW ≤ 2100 g or GA ≤ 33w	34 (4.67)	1 (0.14)	35 (4.81)	96.3	98.7	94.29	98.95

The sensitivity, specificity, positive and negative predictive value of China ROP guideline were 92.3%, 98.3%, 92.30% and 98.32%, respectively. Compared with the proposed screening criteria, the screening criterion of BW ≤ 2100 g or GA ≤ 33 weeks showed maximum the sensitivity, specificity, positive and negative predictive values (96.3%, 98.7%, 94.29% and 98.95, respectively). Retinopathy of prematurity screening criterion of BW ≤ 2100 g or GA ≤ 33 weeks seemed to be the most comprehensive and effective in Tianjin.

## Discussion

Retinopathy of prematurity is a major cause of childhood blindness. Otherwise, the World Health Organization Vision 2020 program defines ROP as an "avoidable disease" [1]. After significant improvements in child healthcare in China, early ROP screenings and prompt treatments decreased the blindness rate of ROP.

The incidence and treatment outcome of ROP in the world are affected by social factors such as economic development, population composition, and the healthcare level of premature infants. With the establishment and improvement of ROP screening and treatment schedules in developed countries, the incidence of unfavorable outcomes due to ROP has been declining. On the other side, the survival rate of very low BW premature infants and critically ill infants has been increasing in developing countries, but healthcare development for newborns has been lagged. These unbalance problems resulted in a higher incidence of ROP in developing countries. In China, the implementation of ROP screening and detection rates vary greatly in different regions. The incidence of ROP in the developed regions showed lower than that in the developing regions [8], more mature premature infants showed a trend to suffer from more severe ROP. The incidence of ROP in the USA increased from 14.70% in 2000 to 19.88% in 2012 [9]. Incidence of ROP was elevated due to the increased survival rate of very preterm infants (GA < 28 W), very low BW infants (BW < 1500 g) and the infants with serious systemic illness who need high concentration oxygen intervention [10].

The incidence of ROP in Beijing, China was 13.1% and the treatment rate was 1.7% in 2012 [11]. The incidence of ROP in Shenzhen was 21%, the treatment rate was 2.2% in 2020 [12]. The incidence of ROP in Shanghai was 17.8%, and the treatment rate was 6.8% in 2013 [13]. A recent study reported that the incidence of ROP in Shanghai was 15.9%, and the treatment rate was 1.1% in 2021 [14]. Tianjin Eye Hospital is the only medical institutions to carried out the ROP screening in Tianjin. Therefore, this study could show the current profiles of ROP in Tianjin. The incidence of ROP (17.9%) and treatment rate (1.9%) in Tianjin were similar to those in other developed regions of China. Otherwise, there were few infants with BW ≤ 750 g or GA ≤ 26w in the study. And no infants with stage 4 and stage 5 of ROP were detected. These results prompted increased levels of prenatal care, neonatal monitoring, and ROP screening. On the other hand, the low survival rate of premature infants with very preterm birth or very low BW leaded to the study population with relatively higher GA and BW.

This study indicated that the incidence of ROP increased gradually with the lower GA and BW. Gestational age and BW showed significant differences between infants with and without ROP. Gender and multiple births showed no differences. Gestational age and BW were still the most important risk factors for ROP. The infants with GA ≥ 35 W showed a relatively high incidence of ROP (3.8%), but most infants suffered from mild ROP. The incidence of ROP was relatively high in infants with higher GA. But the treatment rate was very low, except for the critically ill infants needing oxygen supplements, et al.

The appropriate and effective screening criteria should minimize the number of infants and times of ROP screening. Furthermore, no infants with severe ROP are missed diagnosed. The ROP screening criteria require both accuracy and sensitivity. Retinopathy of prematurity screening guidelines varies over time in different countries based on economic development, population composition and medical level. American Ophthalmological Society has updated the ROP screening guidelines in 2006 [15], 2013 [16], and 2018 [4]. Gestational age, BW, follow-up period, and treatment principles were renewed in different visions of ROP screening guidelines. Economic factors play a major factor role in the incidence and severity of ROP. World Health Organization reported that the mean BW of infants with severe ROP was 750 g in developed countries but 1500 g in developing countries [17]. Fundus examinations of infants with GA > 32w are not recommended according to the ROP screening guidelines of developed countries [4, 18, 19]. A 10-year retrospective study in Brazil reported that 8 infants with GA > 32w and BW > 1500 g were diagnosed with stage 1 of ROP [20]. Ugurbas et al. [21] evaluated the effectiveness of USA and UK ROP screening criteria in Turkish premature infants. One infant with stage 1 of ROP was missed diagnosed according to the USA ROP screening guideline in 2006 (BW ≤ 1500 g or GA ≤ 32w). Two infants with stage 1 of ROP and 2 infants with stage 2 of ROP were missed diagnosed according to the USA ROP screening guideline in 2013 (BW ≤ 1500 g or GA ≤ 30w). Two infants with stage 1 of ROP were missed diagnosed according to the UK ROP screening guideline. Six infants with ROP were missed diagnosed the USA ROP screening guideline in 2001 (BW ≤ 1500 g or GA ≤ 28w), including 2 infants with stage 3 plus lesions located in zone II and III. Romo-Aguas et al. [22] analyzed ROP screening data of 503 infants in Mexico. According to the USA ROP screening guideline in 2013 (BW ≤ 1500 g or GA ≤ 30w), 100 infants were excluded from the ROP screening program, and 57 infants with ROP were missed diagnosed. Of those 15 infants with severe ROP required treatment. Using the ROP screening guideline of the Mexican Secretary of Health in 2015 (GA ≤ 34w or/and BW ≤ 1750 g), 6 infants (1.2%) would be left out of the screening guideline, and 2 infants (33.3%) were diagnosed as ROP and no infants required ROP treatment.

Yang et al. [14] analyzed the screening data of infants from 2012 to 2016 who underwent ROP examinations in four tertiary neonatal intensive care units in Shanghai, China. Using the screening criteria of GA < 32w or BW < 1600 g, 98.4% of infants with type 1 ROP were correctly predicted; meantime, 43.2% of total infants did not require ROP examinations. Only one infant with type I ROP would have been missed. This premature baby was diagnosed with serious systemic diseases. Therefore, the infant should have been identified and screened as an outlier case.

Most screening guidelines were drawn up based on GA and BW which are the identified risk factors of ROP. Other factors, such as oxygen therapy, serious systemic diseases, and long-term hospitalization were reported to correlate to ROP [23]. The appropriate ROP screening criteria should minimize the number of screening infants and examination times, without missing any high-risk cases. Based on the results of this study, some infants with severe ROP were missed diagnosed according to the USA or UK ROP screening guidelines. Two infants with severe ROP were missed diagnosed according to the current China ROP screening guideline. Based on the screening data of Tianjin, we analyzed the sensitivity and effectiveness of different screening criteria (Data was shown in Table 5). If the ROP screening criteria of GA ≤ 33w or/and BW ≤ 2100 g were applied, only one infant with severe ROP was missed diagnosed. This infant had a poor systemic condition and received oxygen therapy. Therefore, the ROP screening criteria of GA ≤ 33w or/and BW ≤ 2100 g are more appropriate and effective in Tianjin.

There are some limitations to this study. Tianjin is one of the most developed cities in China, and the results of this study could not be comprehensive and appropriate for the developing regions of China. Some premature infants did not undergo the fundus examination, because their parents or guardians knew little about ROP. Only data of infants undergoing ROP screening was collected and analyzed, which could introduce bias into the study design. Optimal screening criteria should encompass all infants. However, the most appropriate screening criteria of GA ≤ 33w or/and BW ≤ 2100 g still led to the missing of one infant with severe ROP.

## Conclusion

Gestational age and BW are still major risk factors for the incidence and severity of ROP. And the incidence and treatment rate of ROP in Tianjin is similar to those reported in the other regions of China. Updating the ROP screening criteria and examination schedule would benefit ROP screening and management in Tianjin or China.

## Data Availability

All the data supporting our findings is contained within the manuscript.

## Abbreviations

ROP: Retinopathy of prematurity
BW: Birth weight
GA: Gestational age
USA: United States of America
UK: The United Kingdom of Great Britain and Northern Ireland
AP-ROP: Aggressive posterior retinopathy of prematurity
ETROP: Early Treatment for Retinopathy of Prematurity
VEGF: Anti-vascular endothelial growth factor

## References

[1] Gilbert C, Foster A (2001). Childhood blindness in the context of VISION 2020–the right to sight. Bull World Health Organ.

[2] Zhu JF, Zou HD, He XG (2012). Cross-sectional investigation of visual impairing diseases in Shanghai blind children school. Chin Med J (Engl).

[3] Li  XX (2014). Chinese Ophthalmological Society Fundus Diseases Group. Screening guidelines of retinopathy of prematurity in China (2014). Chin J Ophthalmol.

[4] Fierson  WM (2018). American Academy of Pediatrics Section on Ophthalmology; American Academy of Ophthalmology; American Association for Pediatric Ophthalmology and Strabismus; American Association of Certified Orthoptists. Screening examination of premature infants for retinopathy of prematurity. Pediatrics.

[5] Wilkinson AR, Haines L, Head K (2008). UK retinopathy of prematurity guideline. Early Hum Dev.

[6] International Committee for the Classification of Retinopathy of Prematurity (2005). The International Classification of Retinopathy of Prematurity revisited. Arch Ophthalmol.

[7] Group ETFR (2003). Revised indications for the treatment of retinopathy of prematurity: results of the early treatment for retinopathy of prematurity randomized trial. Arch Ophthalmol.

[8] Wang L, Zhang ZF, Tao MZ (2021). The prevalence of retinopathy of prematurity in the mainland of China from 2008 to 2018. Chin J Ophthalmol.

[9] Ludwig CA, Chen TA, Hernandez-Boussard T (2017). The epidemiology of retinopathy of prematurity in the United States. Ophthalmic Surg Lasers Imaging Retina.

[10] Raghuveer TS, Zackula R (2020). Strategies to prevent severe retinopathy of prematurity: a 2020 update and meta-analysis. NeoReviews.

[11] Li Q, Wang Z, Li Y (2012). Retinopathy of prematurity screening in 2185 premature infants. Chin J Ophthalmol.

[12] Huang HB, Chen YH, Wu J (2020). Early risk factors for retinopathy of prematurity in very and extremely preterm Chinese neonates. Front Pediatr.

[13] Xu Y, Zhou X, Zhang Q (2013). Screening for retinopathy of prematurity in China: a neonatal units-based prospective study. Invest Ophthalmol Vis Sci.

[14] Yang Q, Zhou X, Ni Y (2021). Optimised retinopathy of prematurity screening guideline in China based on a 5-year cohort study. Br J Ophthalmol.

[15] Section on Ophthalmology American Academy of Pediatrics, American Academy of Ophthalmology, American Association for Pediatric Ophthalmology and Strabismus (2006). Screening examination of premature infants for retinopathy of prematurity. Pediatrics.

[16] Fierson  WM, American Academy of Pediatrics Section on Ophthalmology, American Academy of Ophthalmology, American Association for Pediatric Ophthalmology and Strabismus, American Association of Certified Orthoptists (2013). Screening examination of premature infants for retinopathy of prematurity. Pediatrics.

[17] World Health Organization (2000). Preventing Blindness in Children: Report of WHO/IAPB Scientific Meeting.

[18] Wilkinson AR, Haines L, Head K (2008). UK retinopathy of prematurity guideline. Early Hum Dev.

[19] Binenbaum  G, Bell  EF, Donohue  P, G-ROP Study Group (2018). Development of Modified Screening Criteria for Retinopathy of Prematurity: Primary Results From the Postnatal Growth and Retinopathy of Prematurity Study. JAMA Ophthalmol.

[20] Freitas AM, Mörschbächer R, Thorell MR, Rhoden EL (2018). Incidence and risk factors for retinopathy of prematurity: a retrospective cohort study. Int J Retina Vitreous.

[21] Ugurbas SC, Gulcan H, Canan H, Ankarali H, Torer B, Akova YA (2010). Comparison of UK and US screening criteria for detection of retinopathy of prematurity in a developing nation. J AAPOS.

[22] Romo-Aguas JC, González-H León A, Meraz-Gutiérrez MP, Martínez-Castellanos MA (2019). Retinopathy of prematurity: incidence report of outliers based on international screening guidelines. Int J Retina Vitreous.

[23] Hellström A, Smith LE, Dammann O (2013). Retinopathy of prematurity. Lancet.

